# Supplementary figures and images for: High-resolution analysis of human centromeric chromatin
Source: Life Sci Alliance. 2025 Jan 23;8(4):e202402819. doi: 10.26508/lsa.202402819 (PMC11757159; doi:10.26508/lsa.202402819)

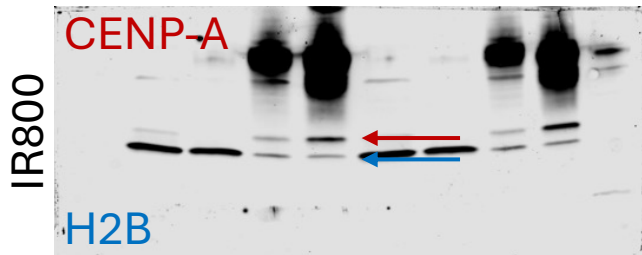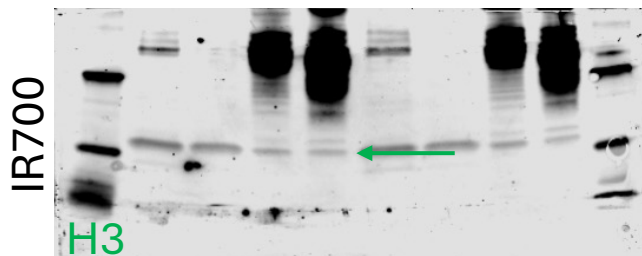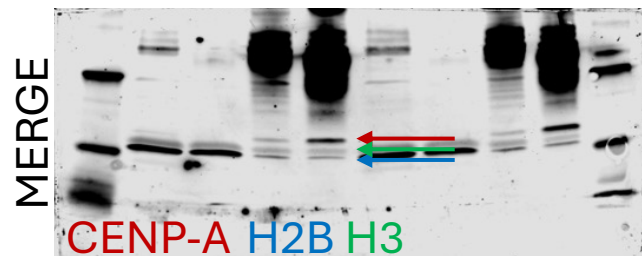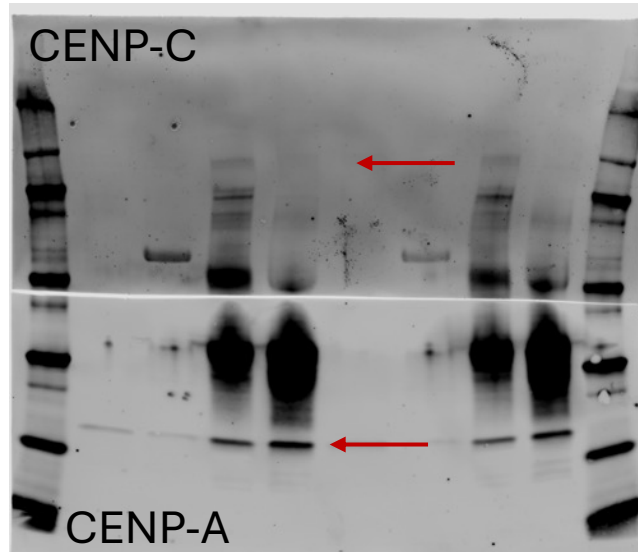

Supplement: Supplementary file 1 [file LSA-2024-02819_SdataF1.1.pdf]

CENP-A

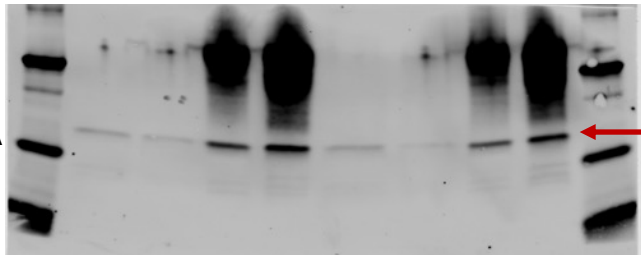

CENP-B

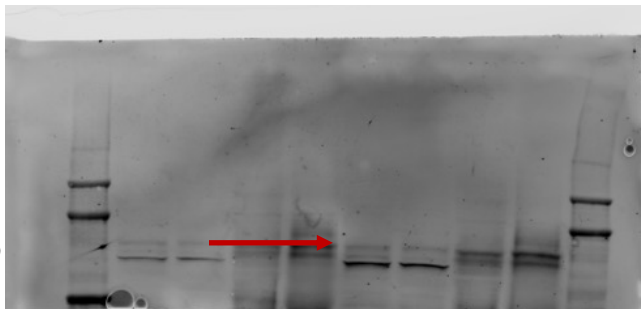

CENP-C

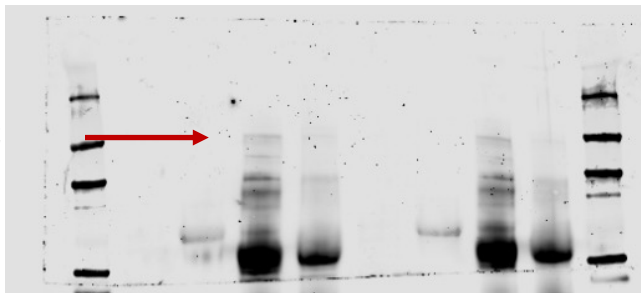

CENP-I

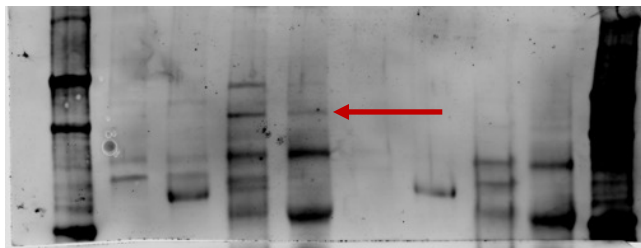

HJURP

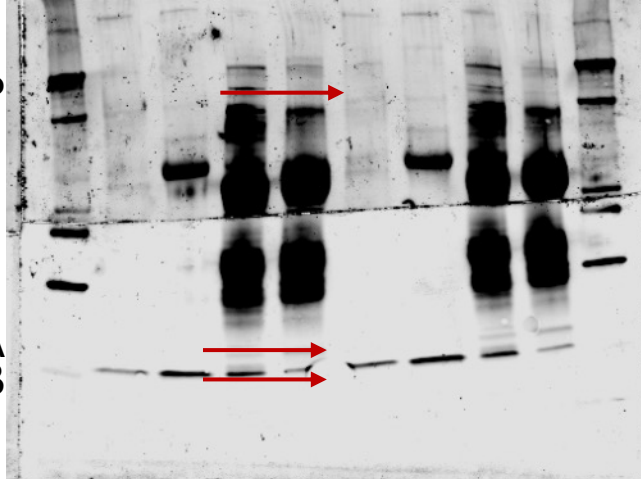

CENP-A  
H2B

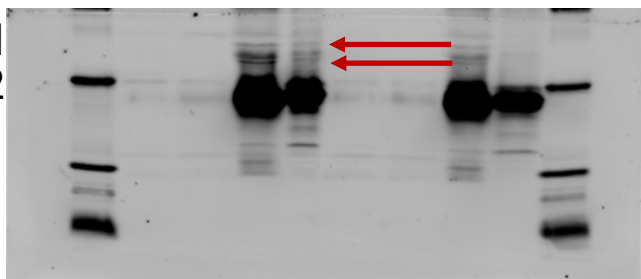

CENP-N  
MIS-12

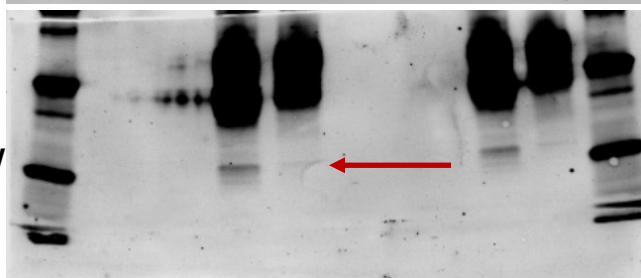

CENP-W

H2A

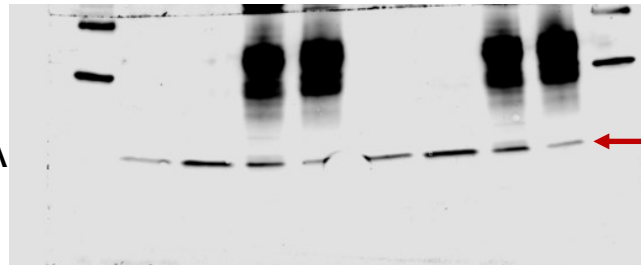

H2B

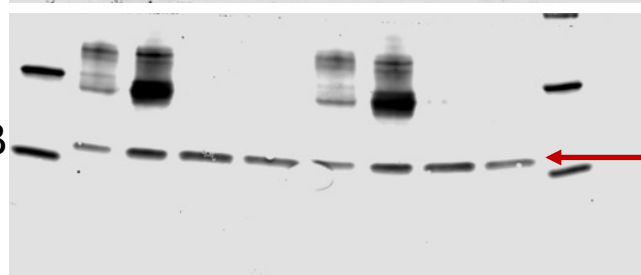

Supplement: Supplementary file 3 [file LSA-2024-02819_SdataF1.3.pdf]

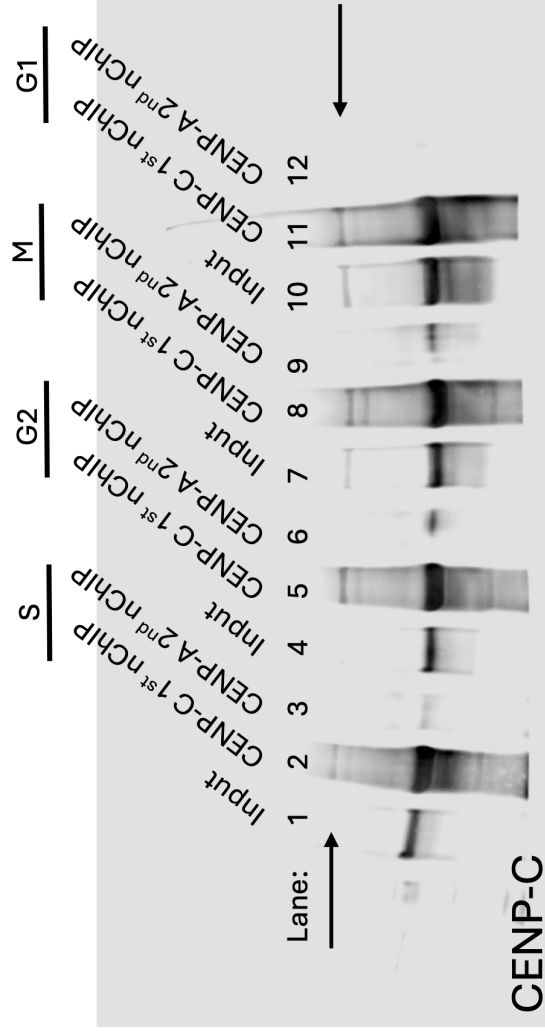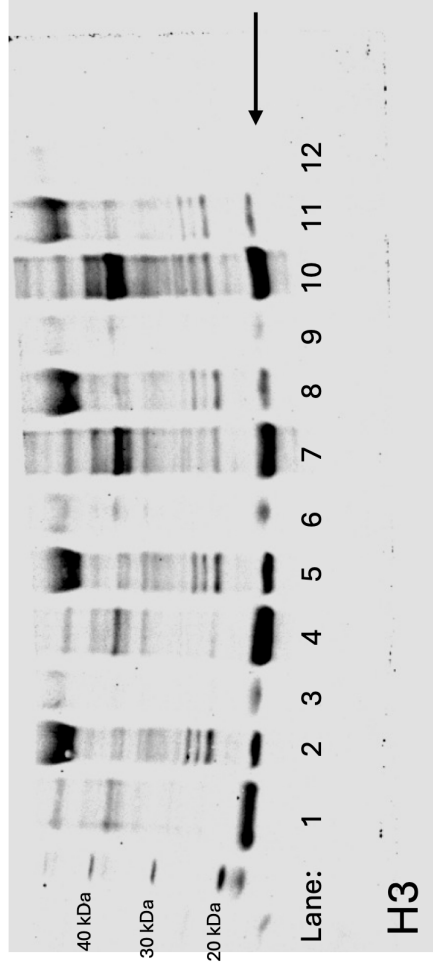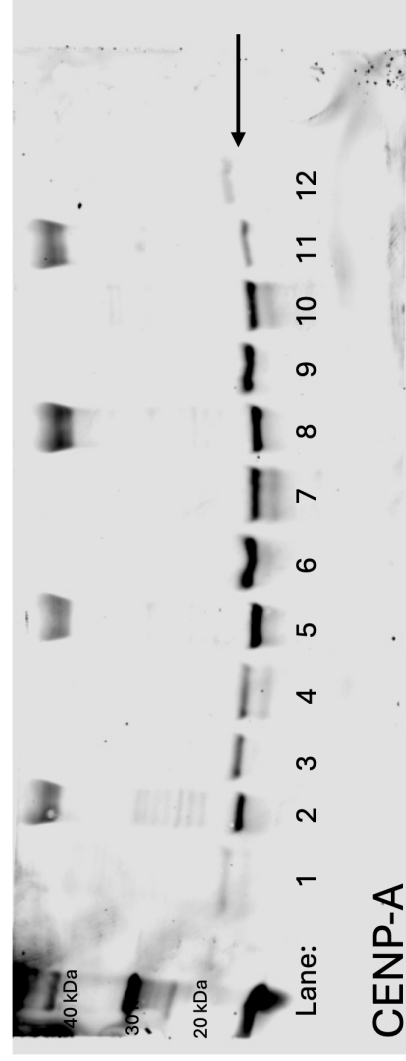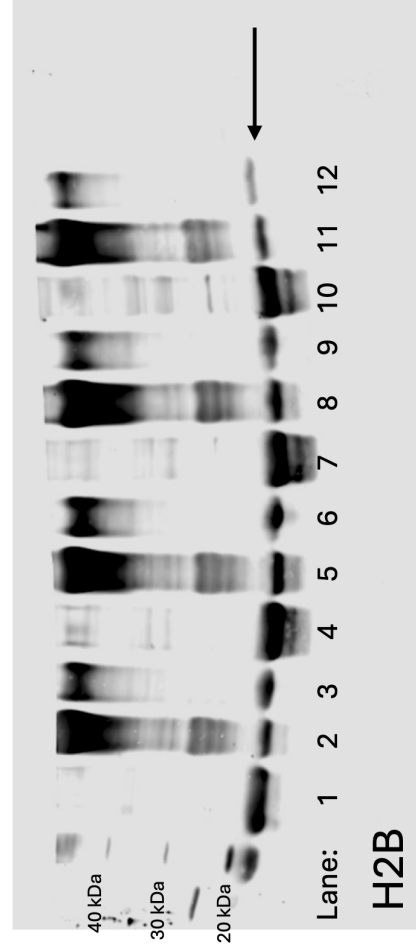

Supplement: Supplementary file 7 [file LSA-2024-02819_SdataF4.1.pdf]

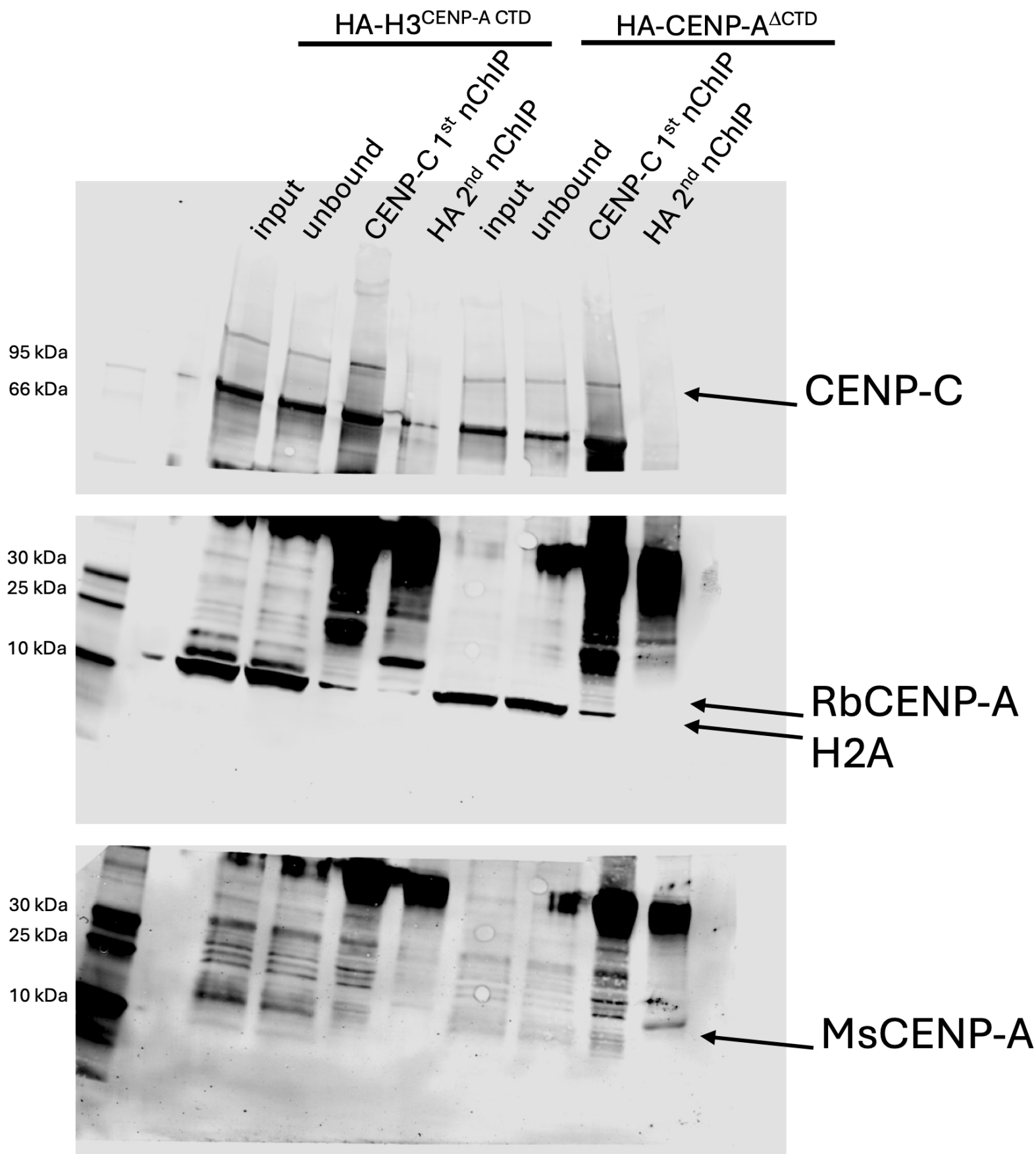

Supplement: Supplementary file 10 [file LSA-2024-02819_SdataF6.2.pdf]

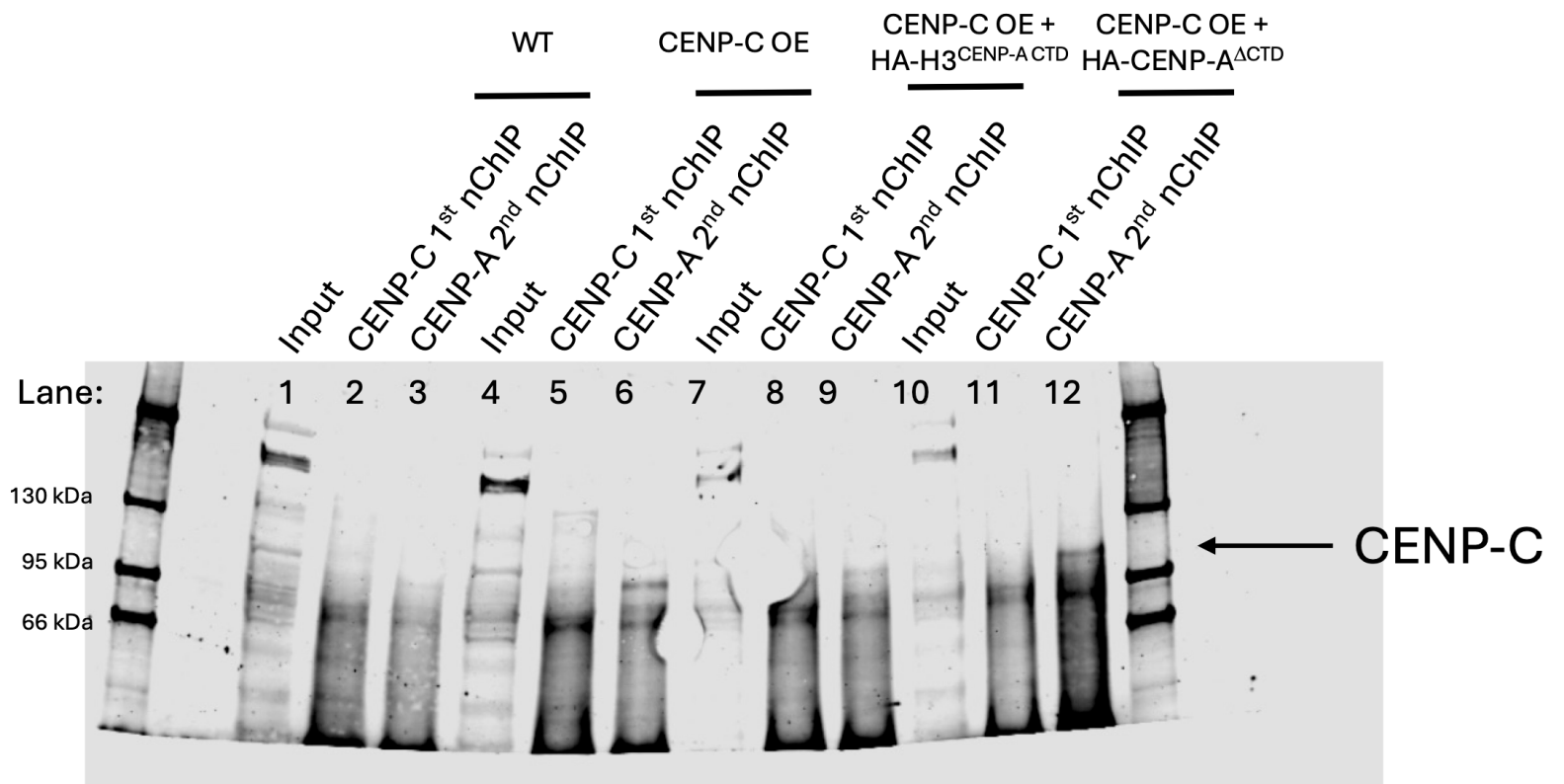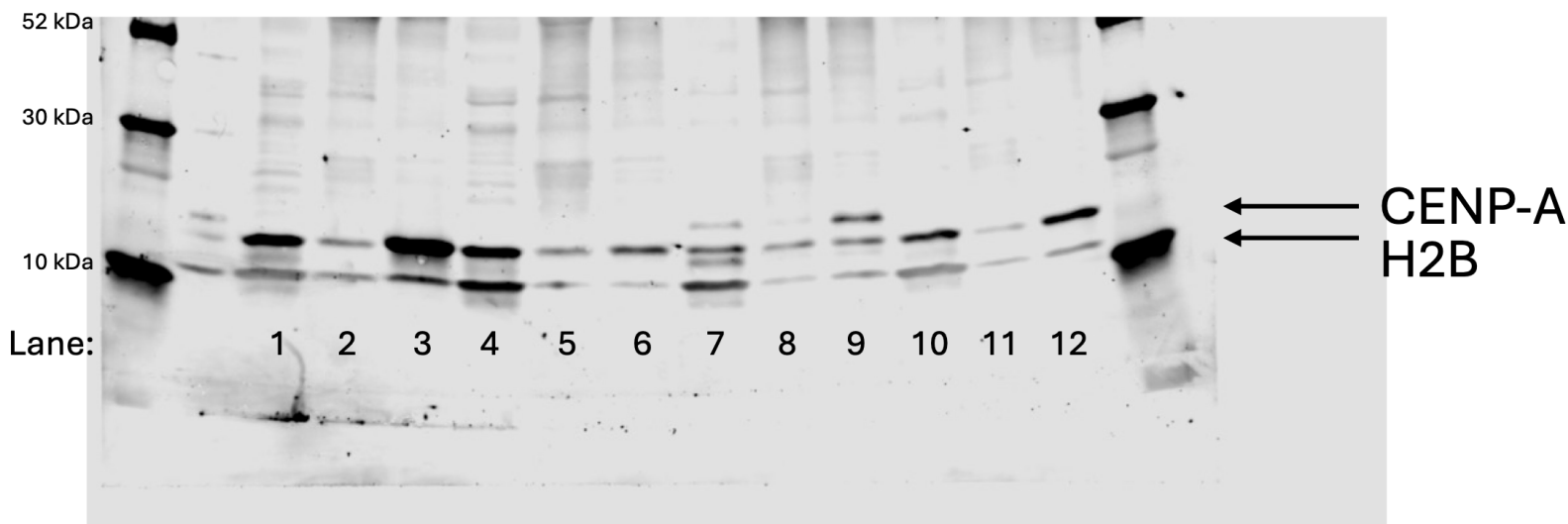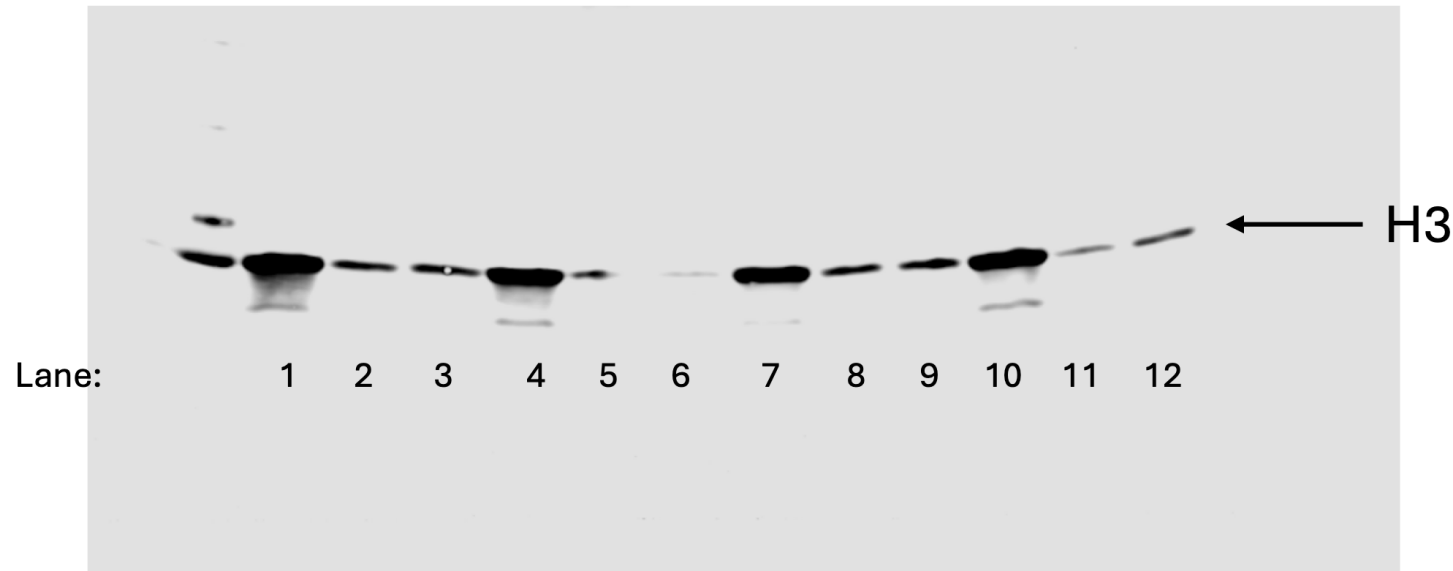

Supplement: Supplementary file 11 [file LSA-2024-02819_SdataF7.1.pdf]
